# Localization of Network-Level Atrophy in Preclinical Alzheimer’s Disease

**DOI:** 10.21203/rs.3.rs-5977523/v1

**Published:** 2025-05-05

**Authors:** Soyoung Lee, Sheena Baratono, Grace Burt, Stephan Palm, William Drew, Benjamin Zide, Nicole Chiulli, Sara Lariviere, Michael Fox, Reisa Sperling, Nancy Donovan, Shan Siddiqi

**Affiliations:** Brigham and Women’s Hospital; Brigham and Women’s Hospital; Brigham and Women’s Hospital; Brigham and Women’s Hospital; Brigham and Women’s Hospital; Brigham and Women’s Hospital; Brigham and Women’s Hospital; Brigham and Women’s Hospital; Brigham and Women’s Hospital; Harvard Aging Brain Study, Department of Neurology, Massachusetts General Hospital, Boston, MA 02114;Center for Alzheimer Research and Treatment, Department of Neurology, Brigham and Women’s; Brigham and Women’s Hospital, Massachusetts General Hospital; Brigham and Women’s Hospital, Massachusetts General Hospital

## Abstract

Brain atrophy may precede symptoms in Alzheimer’s disease (AD), but it remains unclear whether atrophy in this preclinical stage falls within a distinct brain network or is associated with transitional cognitive changes. We investigated cortical thickness in cognitively unimpaired older adults with varying amyloid-β accumulation and estimated the connectivity of each individual’s atrophy pattern using a large normative connectome (n = 1000). A distinct network was connected to atrophy patterns in amyloid-β-positive (n = 1242) versus negative (n = 536) participants. This preclinical AD network was similar to a previously published atrophy network associated with AD dementia (r = 0.8284, p = 0.016). In leave-one-out cross-validation, atrophy patterns connected to the preclinical AD network were associated with lower cognitive performance (p = 0.0018), greater subjective cognitive decline (p < 0.001), and amyloid-β levels (p < 0.001). Atrophy in preclinical AD maps to a network similar to AD dementia that is associated with amyloid-β and cognition, demonstrating an atrophy-related network across the continuum of AD.

## Introduction

Alzheimer’s disease (AD) pathologic changes, including abnormal accumulation of amyloid-β (Aβ), begin many years before individuals develop cognitive and functional impairment. It remains unclear whether Aβ accumulation is associated with a distinct pattern of network-level atrophy during this preclinical stage of the disease. Defining atrophy-related brain networks and clinical manifestations in preclinical AD is critical to understanding initial AD pathophysiology and could lead to better prediction of disease trajectories and potential novel treatment targets in preclinical AD.

Studies of atrophy in preclinical AD have yielded mixed results. In the preclinical stage, it may be difficult to distinguish Aβ-associated atrophy from age-related volume loss or early atrophy from other neurodegenerative conditions, and it emerges later than Aβ and tau pathologies.^1^ Some studies support AD-specific cortical thinning occurs in preclinical AD,^2,3^ while others found no cross-sectional difference^4^ or even increased cortical thickness.^5^ These mixed findings could be due to methodological and technical differences across studies, heterogeneity in the preclinical phase, or inadequate sample size.^6,7^

Mapping lesion-induced symptoms onto the functional brain networks instead of individual brain locations has allowed the localization of various neurologic/psychiatric symptoms.^8^ Recent work suggests that this lesion network mapping technique might be applied to neurodegenerative conditions using atrophy as the “lesion”. Tetreault et al. demonstrated that atrophy locations in individual patients map to brain networks associated with clinical, cognitive, and neuropsychiatric symptoms of AD dementia.^9^ However, this atrophy network mapping (ANM) has never been implemented in preclinical AD, and it is unclear if this method can detect subtle changes in cortical thickness at this stage.

**Figure 1:**
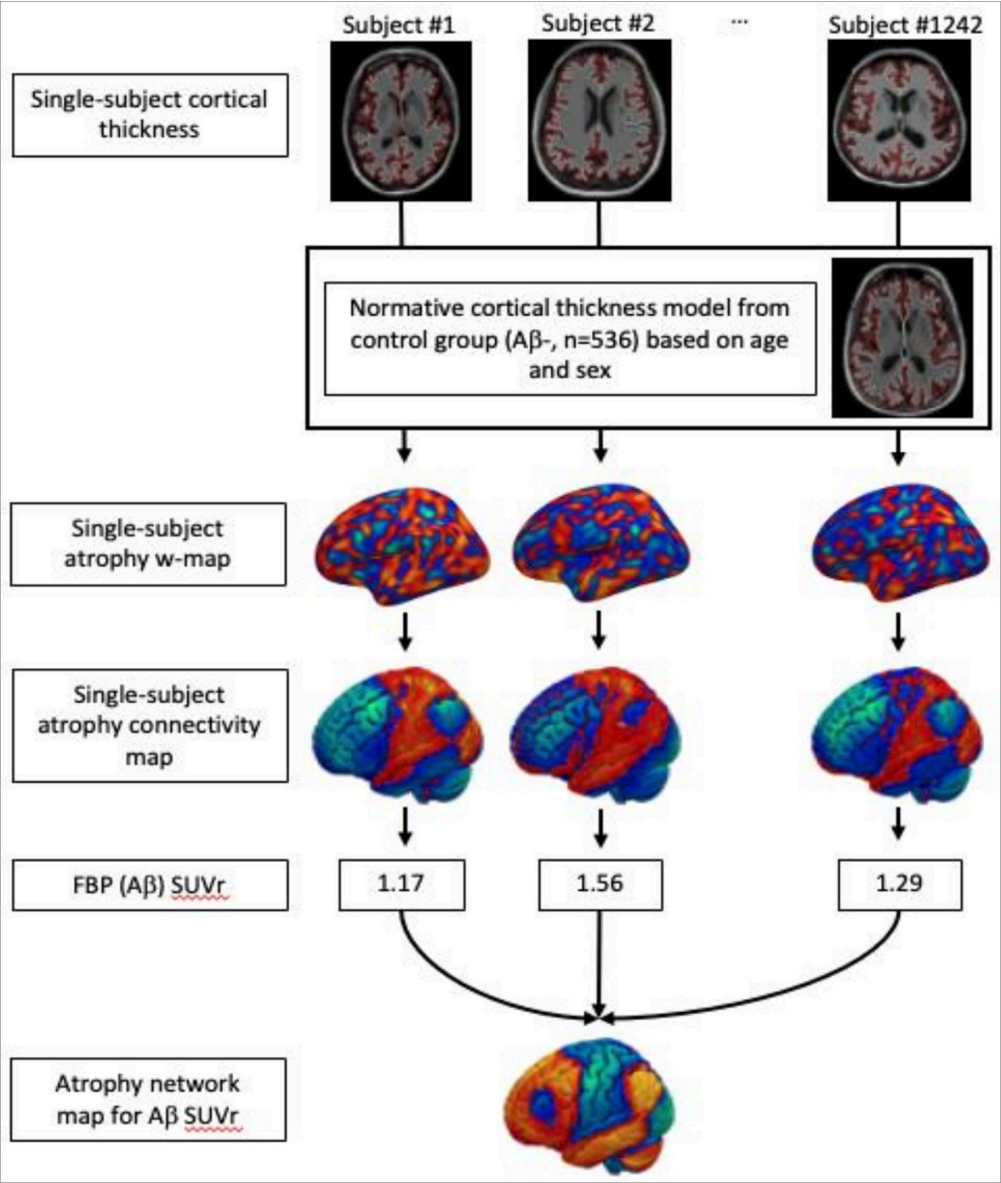
Atrophy Network Mapping. A normative cortical thickness general linear model using Aβ negative subjects as a control group adjusting for age and sex. Comparing to the normative cortical thickness, atrophy w-maps were created for each subject. Then, using the normative functional connectome of the brain, single-subject atrophy connectivity maps were created with each voxel representing the connectivity to atrophy. Finally, comparing atrophy connectivity in each voxel with Aβ SUVr, an atrophy network map for Aβ was created.

This study uses ANM (Fig. 1) to compare network-level neurodegeneration to pathological and clinical manifestations in preclinical AD using a large, well-characterized cohort from an AD prevention clinical trial.^10,11^ We hypothesized that (1) atrophy in the preclinical stage of AD would localize to a distinct brain network, (2) this preclinical AD network would be similar to atrophy patterns previously implicated in AD dementia, and (3) this preclinical AD network would be associated with cognitive manifestations of preclinical AD.

## Results

### Characteristics of the sample

The sample included 1778 older adults from the Anti-Amyloid Treatment in Asymptomatic Alzheimer’s Disease (A4) (ClinicalTrials.gov identifier: NCT02008357) and Longitudinal Evaluation of Amyloid Risk and Neurodegeneration (LEARN) (ClinicalTrials.gov identifier: NCT02488720) studies. A4 was a double-blind, placebo-controlled Alzheimer’s disease prevention trial with anti-amyloid-β (Aβ) therapy in subjects with elevated Aβ accumulation (Aβ+) as determined by ^18^F-florbetapir (FBP) PET scan. LEARN was a companion study of A4 comprised of subjects without high Aβ accumulation (Aβ−).^10,11^ All participants were living independently and determined to be cognitively unimpaired based on a Clinical Dementia Rating global score of 0, a Mini-Mental State Examination (MMSE) score of 25–30, and a Wechsler Memory Scale Logical Memory Delayed Recall score of 6–18. Participants underwent brain imaging, including structural brain MRI and FBP PET.^10,11^ The Aβ status (Aβ + vs. Aβ−) was determined using a dual quantitative and qualitative algorithm, using global FBP-specific binding calculated from six regions (anterior cingulate, posterior cingulate, frontal cortex, temporal cortex, parietal cortex, and precuneus) and visual read. ^10,11^

Demographics and other covariates between the Aβ− and Aβ + groups are summarized in Table 1. The mean (SD) age was 71.5 (4.7) years, 1060 (59.6%) were female, and the mean (SD) education was for 16.6 (2.8) years. Cognitive performance was measured using the Preclinical Alzheimer Cognitive Composite (PACC).^12,13^ Subjective cognitive decline was assessed with the Cognitive Function Index (CFI),^14,15^ which consists of subjective cognitive concerns reported by study partners (CFI-SP) and study participants (CFI-PT).

In comparison to the Aβ− group, the Aβ + group was older (p-value < 0.001, t statistic = −6.704), had lower PACC score (p-value < 0.001, t-value = 5.294), and reported greater CFI score (p-value < 0.001, t-value = −5.129), as reported in prior analyses of the same dataset.^11^ These differences remained significant when adjusting for age, sex, and education. (Table 1)

### Cortical thinning and medial temporal lobe atrophy in Aβ + vs. Aβ−

**Figure 2:**
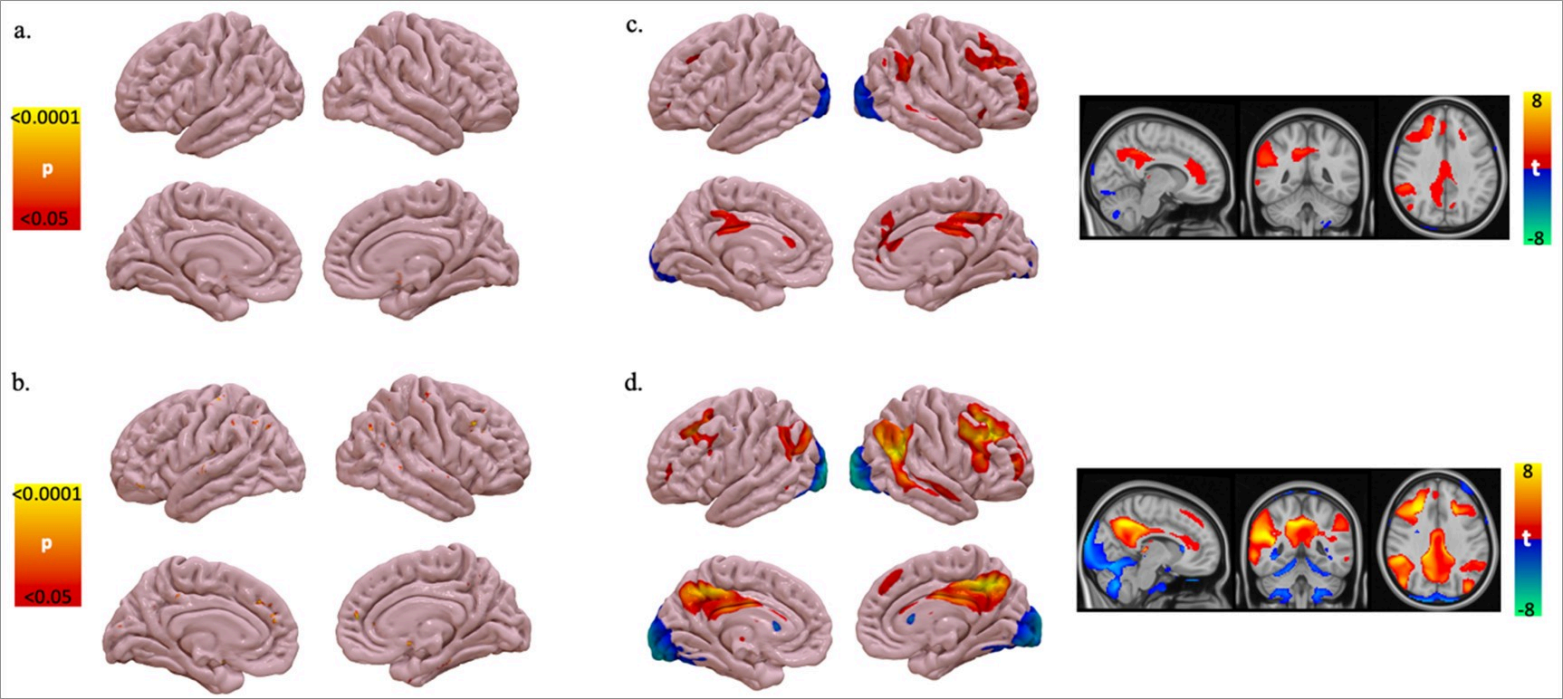
Localizing atrophy in preclinical Alzheimer’s disease. a. Vertex-wise ANOVA of cortical thickness between **A**β**+ Vs. A**β-controlling for age, sex, and education. FWE-corrected p<0.05. b. Vertex-wise regression of cortical thickness with **continuous FBP (A**β**)SUVr** using PALM controlling for age, sex, and education. FWE-corrected p<0.05. c. Atrophy network map for preclinical Alzheimer’s disease: voxel-wise ANOVA of functional connectivity to atrophy between **A**β**+ Vs. A**β-controlling for age, sex, and education. FWE-corrected p<0.05. d. Atrophy network map for preclinical Alzheimer’s disease: voxel-wise linear regression of functional connectivity to atrophy and **continuous FBP (A**β**) SUVr** controlling for age, sex, and education. FWE-corrected p<0.05.

First, we compared traditional metrics of atrophy between Aβ + and Aβ− groups. There was no significant difference between groups in terms of global cortical thickness or adjusted hippocampal volume. These results were unchanged when adjusting for age, sex, and education (Table 1). In a vertex-wise cortical thickness comparison between Aβ + and Aβ− groups, no specific regions remained statistically significant after family-wise error (FWE) correction using the permutation-based max t-stat method as implemented by Winkler et al.^16^ Thus, atrophy location alone was not significantly different between Aβ + and Aβ− groups. (Fig. 2.a)

Next, we compared cortical thickness to continuous Aβ SUVr using voxel-wise Pearson correlation, as continuous scores may be more sensitive than binary classification,^17^ adjusting for age, sex, and education. After FWE correction, higher Aβ SUVr values were correlated with small, scattered clusters of cortical thinning in the superior frontal gyrus, postcentral gyrus, posterior cingulate/precuneus, superior temporal gyrus, parahippocampal gyrus, and cuneus. (Fig. 2.b)

### Atrophy Network Maps for Preclinical AD: Aβ + vs. Aβ− and continuous Aβ SUVr

To test for network-level effects, which may be more sensitive than looking at individual regions of atrophy, we performed ANM according to the previously published method.^9^ We created a general linear model (GLM) for expected cortical thickness controlling for age and sex using 546 Aβ− subjects’ structural MRI. Next, atrophy w-maps were created for each subject, with each vertex representing an atrophy w-score in comparison to the normative cortical thickness from the GLM. Then, we estimated the whole-brain connectivity of each participant’s atrophy pattern using a normative connectome database (n = 1000),^18,19^ as in prior work on lesion and ANM. (Fig. 1)

To map the connectivity of atrophy patterns associated with AD pathology, we used a voxel-wise ANCOVA to compare the whole-brain atrophy connectivity between the Aβ + and Aβ− groups, adjusting for age, sex, and education. The Aβ + group had atrophy patterns with greater connectivity to the right prefrontal lobe, right precuneus, right temporoparietal junction (Fig. 2.c), which remained statistically significant after FWE correction.^16^ Similar results were seen when using continuous Aβ SUVr instead of binarizing Aβ + vs. Aβ−, but more brain regions survived multiple comparisons correction, including right dorsolateral and medial prefrontal lobes, left and right precuneus, left and right lateral parietal lobes, and the right temporoparietal junction and middle temporal gyrus (Fig. 2.d).

**Figure 3:**
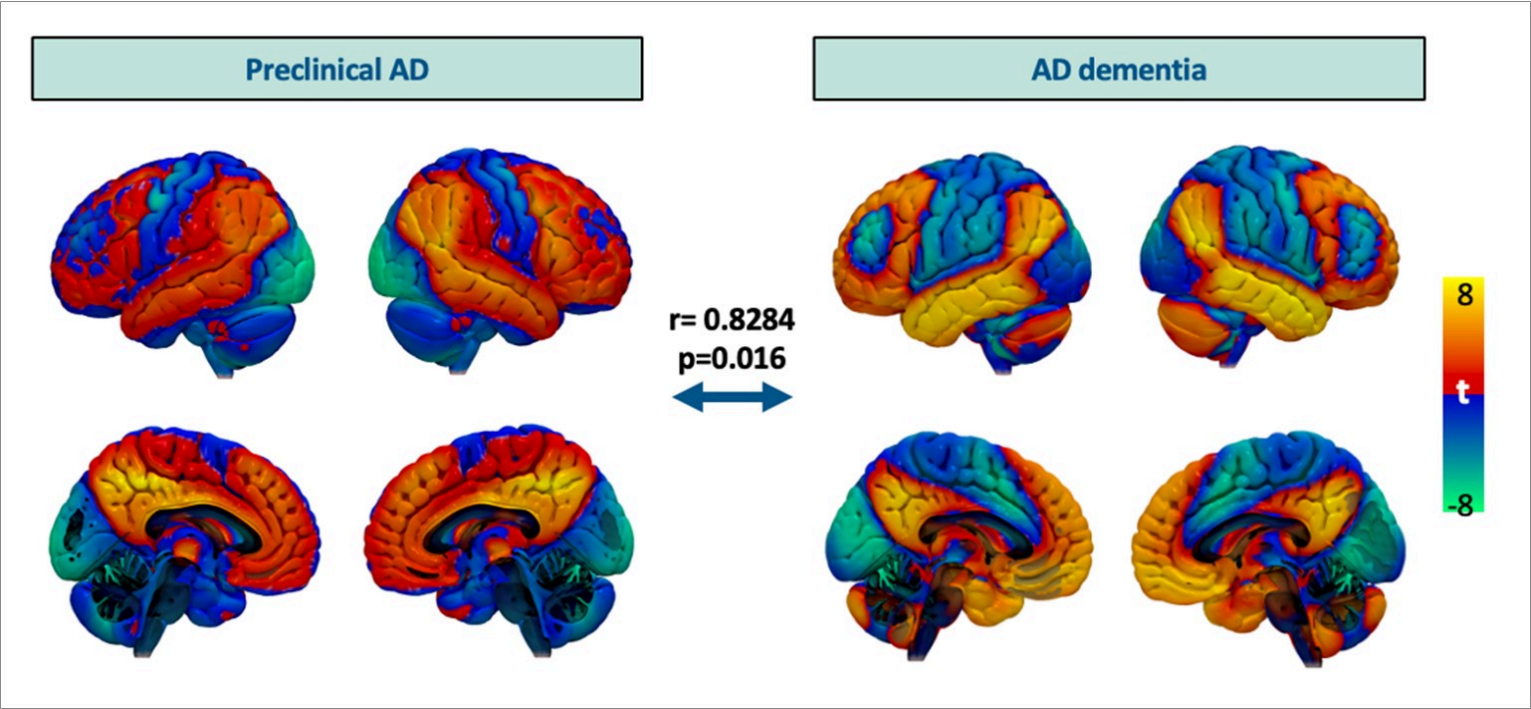
Comparison between atrophy network map for preclinical AD versus pre-established atrophy network map for AD dementia derived from ADNI-2 dataset. Preclinical AD map is based on continuous Aβ SUVr from the A4/LEARN dataset following the atrophy network mapping procedures described in Figure 1. Atrophy network map for AD dementia is created, comparing Aβ ^+^ subjects with AD dementia and Aβ^−^, cognitive normal subjects.

This atrophy network map using Aβ SUVr was compared to the previously published atrophy network map for AD dementia.^9^ This map was created using the data from Alzheimer’s Disease Neuroimaging Initiative II (ADNI-2) following the same procedure that was described above, but comparing patients with clinically adjudicated AD dementia with high Aβ to those who were deemed cognitively normal with low Aβ. The two maps were more similar to each other than by chance (r = 0.8284, p = 0.016) using permutation testing in which a spatial correlation was recomputed after each patient’s clinical measure was randomly permuted against a different patient’s neuroimaging, as in prior work.^20^ Thus, atrophy associated with Aβ positivity in preclinical AD localized to a similar network as atrophy associated with a clinical diagnosis of AD dementia. (Fig. 3)

Atrophy Network Maps for Preclinical AD is associated with cognition.

**Figure 4.**
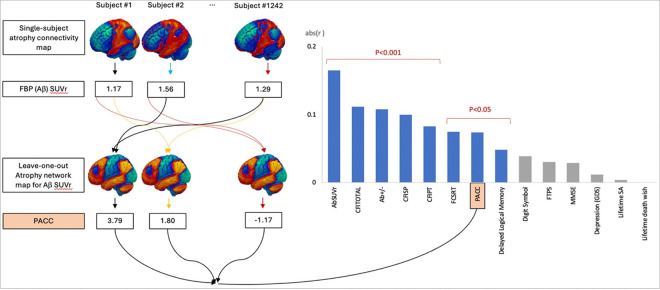
Leave-one-out cross-validation: spatial correlation between individual atrophy connectivity map and leave-one-out atrophy network map for preclinical AD was associated with individual subjects’ Aβ SUVr, dichotomous Aβ+/− group, cognition measured by PACC and CFI, but not with some of PACC subdomains and behavioral variables including depression. PACC=Preclinical Alzheimer Cognitive Composite, Digit Symbol= Digit Symbol Substitution Test score from the Wechsler Adult Intelligence Scale–Revised, Logical Memory Delay= Logical Memory IIa sub-test from the Wechsler Memory Scale, FCSRT= Free and Cued Selective Reminding Test, MMSE=Mini-Mental Status Exam, CFI=Cognitive Function Inventory, SP=Study Partner, PT=Participant. FTPS= Future Time Perspective Scale, GDS= Geriatric Depression Scale, SA= suicidal attempt.

Next, we tested whether the Aβ SUVr atrophy network map could explain variance in clinical factors in an out-of-sample analysis, using a leave-one-out cross-validation. First, the Aβ SUVr atrophy network map was regenerated after excluding one participant. The resulting map was compared to the left-out map using spatial Pearson correlation. This leave-one-out spatial correlation was computed for each participant. (Fig. 4) As expected, atrophy to the preclinical AD network was associated with A *β* status in this out-of-sample analysis (r = 0.11, p < 0.001). Similarly, atrophy to the preclinical AD network was associated with Aβ SUVr (r = 0.17, p<0.001).

The resulting spatial correlations were also compared with clinical manifestations, including objective cognitive performance measured by PACC and subjective cognitive decline measured by CFI total score, using Pearson correlations. (Fig. 4). The leave-one-out correlation was negatively associated with PACC scores (r= −0.0740, p = 0.0018) and positively associated with CFI total score (r = 0.1119, p < 0.001). The leave-one-out correlation was also associated with specific subdomains of the PACC, including the Digit Symbol Substitution Test (Digit Symbol), Free and Cued Selective Reminding Test (FCSRT), and Mini-Mental Status Exam (MMSE), but not Logical Memory IIa sub-test from the Wechsler Memory Scale (Delayed Logical Memory). The leave-one-out correlation was also significantly associated subjective cognitive decline as detected by both the CFI participant report and the CFI study partner report. In contrast, the leave-one-out correlation was not associated with other nonspecific behavioral variables, including depression measured by the Geriatric Depression Scale, optimism measured by the Future Time Perspective Scale, history of lifetime suicidal attempt, and history of lifetime death wishes.

## Discussion

Atrophy network mapping revealed a discrete and localizable pattern of atrophy in preclinical AD, using a well-characterized, large cohort of cognitively unimpaired older adults in AD prevention studies.^10,11^ This pattern was tested against the gold standard of Aβ PET neuroimaging showing a significant association. This network was similar to the previously established atrophy network for AD dementia.^9^ Moreover, similarity of given subjects’ atrophy connectivity to this preclinical AD network was associated with transitional objective and subjective cognitive manifestations in the unimpaired subjects. These results add to the validity of the atrophy network map in preclinical AD. Furthermore, they indicate that network-level atrophy is already detectable during this initial biological stage of AD and is associated with early cognitive manifestations.

Understanding AD neuropathology, which begins to accrue years before symptoms arise, has paved the way for developing disease-modifying and potential disease-preventing therapies.^10,21–23^ This endeavor resulted in a biological definition of AD based on elevated neuropathological biomarkers even in individuals without overt cognitive and functional decline: preclinical AD.^24^ However, some have questioned whether this unimpaired stage should be considered a distinct disease entity.^25,26^ Here, we demonstrated that AD-specific neurodegeneration is detectable in preclinical AD by localizing atrophy connectivity in cognitively unimpaired older adults with Aβ. This confirms that the preclinical stage is associated not only with AD proteinopathies, but also with structural neurodegeneration. This supports that preclinical AD, defined by Aβ accumulation, is not a risk factor but rather an early part of the AD disease process.

Investigating atrophy in AD has faced challenges, partly due to its high individual variability. By applying network mapping to atrophy in Alzheimer’s disease, Tetreault et al. elucidated the localization of clinical, cognitive, and psychotic symptoms of AD dementia. These network-level patterns explained more variance than any individual regions of atrophy. However, the relevance of atrophy to preclinical AD, the stage of the AD continuum prior to impairment, has remained unclear.^1^ In this study, we demonstrated that atrophy network mapping could be used in preclinical AD to localize the atrophy connectivity patterns at this stage, which was similar to Tetreault’s AD dementia network. This validates the use of atrophy network mapping in preclinical AD, potentially broadening the use of this method to study other symptoms in preclinical AD datasets.

To our knowledge, this is the first study demonstrating a network-specific pattern of atrophy connectivity and its associations with cognition in preclinical AD. Leave-one-out atrophy network maps showed that the similarity of a given subject’s atrophy connectivity to the preclinical AD network is significantly associated with poor objective cognition and greater subjective cognitive decline. This was specific to cognitive manifestations relative to other behaviors associated with AD such as depression, suggesting a relevance of the preclinical AD network to cognitive changes characteristic of AD.

The atrophy network for preclinical AD included the right dorsolateral and medial prefrontal lobe, left and right precuneus, left and right lateral parietal lobes, and right middle temporal gyrus, key areas of the default mode network that are affected in Alzheimer’s disease.^27,28^ Interestingly, the medial temporal lobe did not survive correction for multiple comparisons. This could be explained by selection bias, that subjects with atrophy connected to the medial temporal lobe may more likely be symptomatic, and thus would have been excluded from the study.^29^ The asymmetry of this network could be due to a similar selection bias, as subjects with left cortical involvement may be more likely to progress to a symptomatic stage due to specific cognitive deficits related to language or verbal memory.^30^

Of note, the effect sizes detected in this study are relatively small. There are several sources of variability that may contribute to this. One key factor is that this was a cross-sectional study rather than a longitudinal study, so patients are being compared to one another rather than to their own baseline, which limits the explainable variance. Atrophy and cognition can be quantified more precisely when comparing a subject to their own prior scores. Also, this study analyzed subjects in preclinical AD solely based on Aβ without consideration of heterogeneity due to other factors, e.g. tau, that play a role in AD pathogenesis in this stage. While this could introduce variability, this study design represents a real-world population and is consistent with the latest revised criteria for diagnosis and staging of Alzheimer’s disease.^24^ There are also additional sources of noise that limit explainable variance; for instance, even though Aβ PET is now considered a gold standard, it only explains 50–60% of the variance in pathological Aβ levels.^31^ These factors are offset by the large sample size, which enabled us to detect a significant effect despite multiple sources of variance. For these reasons, the present analysis is informative to better understand the disease process, but future longitudinal studies would be necessary before considering its use for clinical diagnostic purposes. Other limitations include the use of a normative connectome database derived from healthy, young controls, which may not reflect the brain connectivity changes in AD or older adults. However, prior work suggests that the signal-to-noise benefit of a large connectome exceeds minor differences in patient- or disorder-specific connectivity.^32^

Overall, using ANM, we identified a distinct atrophy connectivity pattern in preclinical AD, supporting the notion that preclinical AD is part of the AD disease process with ongoing neurodegeneration.

## Online Methods

### Subjects

The cross-sectional clinical and neuroimaging data from cognitively unimpaired (CU) older adults were obtained from the publicly available Anti-Amyloid Treatment in Asymptomatic Alzheimer’s Disease (A4, ClinicalTrials.gov identifier: NCT02008357) and Longitudinal Evaluation of Amyloid Risk and Neurodegeneration (LEARN, ClinicalTrials.gov identifier: NCT02488720) dataset (https://ida.loni.usc.edu/login.jsp). The A4 study was a double-blind, placebo-controlled, secondary prevention trial testing whether the anti-amyloid antibody solanezumab could slow cognitive decline in CU older adults with elevated brain Amyloid-β (Aβ). The LEARN study, an observational companion study, followed individuals without elevated brain Aβ who were excluded from the A4 study. Screening procedures have been previously described.^1,2^ Eligible participants were ages 65–85, living independently, and cognitively unimpaired based on a Clinical Dementia Rating global score of 0, a Mini-Mental State Examination (MMSE) score of 25–30, and a Wechsler Memory Scale Logical Memory Delayed Recall score of 6–18. Exclusion criteria included a diagnosis of cognitive impairment or dementia, the use of AD medications, unstable medical conditions, or significant anxiety or depression that could pose a risk for disclosing a participant’s Aβ imaging results. The local Institutional Review Board approved the A4/LEARN studies at each of the clinical trial sites. All participants provided written informed consent prior to screening.

### Demographic Information

The data included demographic information including age, sex, and education.

### Objective Cognition

Cognitive performance in the A4/LEARN studies was measured using the Preclinical Alzheimer Cognitive Composite (PACC).^3,4^ The PACC score was computed as the mean *z* score from the Logical Memory Delayed Recall (Logical Memory), the MMSE, the Weschler Adult Intelligence Scale-Revised Digit Symbol Coding (Digit Symbol), and the Free and Cued Selective Reminding Test (FCSRT). A higher PACC *z* score denotes better cognitive functioning.

### Subjective Cognitive Decline

Subjective cognitive decline was assessed with the Cognitive Function Index, a 15-item survey administered individually to the participant and their study partner that asks about changes in the participant’s cognitive function over the last year.^5,6^ Respondents chose “yes”, “no”, or “maybe” for each question. A total score was derived by summing the participant’s and study partner’s item scores, with a higher score indicating greater subjective cognitive decline.

### Other Covariates

Depressive symptoms were measured by the 15-item version of the Geriatric Depression Scale,^7^ and perception of one’s future was measured by the 10-item Future Time Perspective Scale.^8^ The data also included a history of lifetime suicidal attempts and a history of lifetime passive death wishes.

### Structural MRI Acquisition and Freesurfer Processing

3T brain structural MRI was obtained on study-qualified MRI scanners. T1 images were processed using Freesurfer 6.0.^9^

### Global Cortical Thickness

Global cortical thickness was calculated by averaging the cortical thickness across all cortical vertices from cortical thickness output from Freesurfer 6.0^9^’s recon-all process.

### Hippocampal Occupancy Score

The hippocampal occupancy was calculated as the ratio of hippocampal volume to the sum of the hippocampal and inferior lateral ventricle volumes in each hemisphere, left and right hippocampal occupancy scores were averaged, and then normalized for age and sex.^10^

### ^18^F-florbetapir PET Imaging

All participants underwent Aβ PET using ^18^F-florbetapir (FBP).^1^ Aβ PET was acquired from 50 to 70 minutes after a 10mCi bolus injection and was reconstructed in 4x-5minute frames. Global FBP specific binding was calculated from six regions (anterior cingulate, posterior cingulate, frontal cortex, temporal cortex, parietal cortex, and precuneus) as a mean standardized uptake value ratio (SUVr) normalized to a whole-cerebellum reference region.^11^ Aβ status (Aβ+ vs. Aβ−) was determined using a dual quantitative and qualitative method. An SUVr of 1.15 or greater was the primary criterion for Aβ+ status.^12^ An SUVr between 1.10 and 1.15 was considered Aβ+ if a visual read confirmed positivity.

### Statistical Analyses

Statistical analyses were completed using Matlab R2022b. The global cortical thickness and hippocampal occupancy score of Aβ+ group was compared against Aβ− group using ANCOVA with age, sex, and education as covariates. The vertex-wise cortical thickness was compared between Aβ+ and Aβ− groups with age, sex, and education as covariates, then evaluated using regression models by treating Aβ SUVr as a continuous variable adjusting for the same covariates. For the vertex-wise analysis, the p-value was corrected for multiple comparisons family-wise error correction using the permutation-based max t-stat method implemented by Winkler et al.^13,14^

### Atrophy Network Mapping (ANM)

ANM was performed following the previously published procedure by Tetreault et al.^15^ (Figure 1) We created a vertex-wise general linear model (GLM) for cortical thickness for the cognitively unimpaired subjects without Aβ accumulation (Aβ−) using age and gender as covariates. Next, we calculated a vertex-wise w-score for cortical thickness in each patient (a w-score is a z-score adjusted for covariates, in this study, age and sex). Next, we derived an ‘atrophy connectivity map’ for each subject, defined as the brain regions functionally connected to each subject’s atrophy w-map. First, single-subject atrophy w-maps in surface space from each hemisphere were combined and converted to MNI volume space. Using a publicly available normative functional connectivity dataset of 1000 healthy subjects from the Genome Superstruct Project (GSP),^16,17^ we computed the average blood oxygen level-dependent (BOLD) time course for all voxels within each patient’s distributed single-subject atrophy w-map. Next, we correlated this mean time course within each single-subject atrophy w-map with the BOLD time course at every other brain voxel.

#### 1) ANM of Preclinical AD Comparing Aβ− vs. Aβ+

We compared atrophy connectivity maps between Aβ− vs. Aβ+ groups at every voxel using ANCOVA, including age, sex, and education as covariates. The resulting atrophy network map was corrected for multiple comparisons by permutation-based family-wise error correction (p < 0.05).^14^

#### 2) ANM of Preclinical AD Using Continuous Global Aβ SUVr

We performed voxel-wise analyses comparing atrophy connectivity maps to each subject’s global Aβ SUVr using a linear regression model adjusting for age, sex, and education. Again, the resulting atrophy network map was corrected for multiple comparisons by permutation-based family-wise error correction (p < 0.05).^14^

#### 3) ANM of AD dementia using Alzheimer’s Disease Neuroimaging Initiative II (ADNI-2)

We replicated ANM of AD dementia in comparison to cognitively normal subjects from ADNI-2 dataset following the published ANM procedure by Tetreault et al.^15^ We defined the control group with cognitive normal subjects with low Aβ and AD dementia group with subjects with AD diagnosis and high Aβ, based on the same Aβ SUVr cut-off as the A4/LEARN studies.

### Random Permutation Testing

To examine similarity of two brain maps, the atrophy network map for preclinical AD from A4/LEARN versus the atrophy network map for AD dementia from ADNI-2, a random permutation testing was performed as in prior work.^18^ Briefly, a spatial correlation was recomputed after each patient’s clinical measure was randomly permuted against a different patient’s neuroimaging. After repeating this permutation 1000 times, the chances of random spatial correlation values being greater than the true spatial correlation between the two maps were calculated.

### Leave-one-out cross-validation

Leave-one-out atrophy network maps for preclinical AD using continuous Aβ SUVr were generated for each subject using the atrophy connectivity maps from the whole sample except for the particular subject. Each subject’s atrophy connectivity map was compared to its leave-one-out atrophy network map by spatial correlation. To examine if the similarity of each subject’s atrophy connectivity to each subject’s leave-one-out atrophy network map for preclinical AD is associated with the given subject’s true Aβ status, t test was performed between the r-value from the spatial correlation and the dichotomous Aβ group. Then, the same leave-one-out analysis was performed for Aβ SUVr by linear regression between the spatial r-value the continuous Aβ SUVr. To examine if the similarity of each subject’s atrophy connectivity to each subject’s leave-one-out atrophy network map for preclinical AD is associated with given subject’s cognition, linear regression was performed for the correlation coefficient as a predictor with objective cognition measured by PACC and subjective cognitive decline measured by CFI. The same analyses were performed for subdomains for PACC, Digit Symbol Substitution Test (Digit Symbol), Free and Cued Selective Reminding Test (FCSRT), and Mini-Mental Status Exam (MMSE), Logical Memory IIa sub-test from the Wechsler Memory Scale (Delayed Logical Memory), subjective cognitive decline reported by study partner (CFI-SP), subjective cognitive decline reported by participant (CFI-PT), depression measured by GDS, future-time perspective scale, history of life-time suicidal attempt, and history of life-time death wish.

## Figures and Tables

**Table 1:** Characteristics of A4/LEARN Baseline Cohort by Amyloid Status

	Total (N = 1778)	Aβ negative (N = 536)	Aβ positive (N = 1242)	p
**Age (Mean [SD])**	71.54 [4.74]	70.46 [4.29]	72.00 [4.84]	< 0.001
**Sex (N of Female [%])**	1060 [59.6%]	328 [61.2%]	732 [58.9%]	0.37
**Education**	16.62 [2.76]	16.76 [2.62]	16.56 [2.81]	0.16
**PACC**	−0.20 [2.63]	0.28 [2.41]	−0.41 [2.69]	0.003
**Digit Symbol**	43.61 [8.93]	44.15 [8.82]	42.59 [8.93]	0.18
**FCSRT**	75.88 [6.04]	76.72 [5.71]	75.52 [6.14]	0.02
**Logical Memory Delay**	11.56 [3.34]	11.89 [3.35]	11.42 [3.32]	0.1
**MMSE**	28.80 [1.24]	28.94 [1.13]	28.75 [1.28]	0.05
**CFI**	7.48 [6.31]	6.12 [5.77]	7.94 [6.42]	< 0.001
**CFI-SP**	3.11 [3.87]	2.53 [3.38]	3.31 [4.00]	< 0.001
**CFI-PT**	4.31 [3.83]	3.58 [3.55]	4.63 [3.91]	< 0.001
**Average Cortical Thickness**	2.18 [0.08]	2.19 [0.08]	2.18 [0.08]	0.23
**Hippocampal Occupancy** *	0.73 [0.08]	0.76 [0.07]	0.73 [0.08]	0.51
**PET_SUVr**	1.23 [0.22]	0.99 [0.07]	1.33 [0.18]	< 0.001

SD = Standard Deviation, PACC = Preclinical Alzheimer Cognitive Composite, Digit Symbol = Digit Symbol Substitution Test score from the Wechsler Adult Intelligence Scale-Revised, Logical Memory Delay = Logical Memory IIa sub-test from the Wechsler Memory Scale, FCSRT = Free and Cued Selective Reminding Test, MMSE = Mini-Mental Status Exam, CFI = Cognitive Function Inventory, SP = Study Partner, PT = Participant.

* Hippocampal Occupancy was calculated as the ratio of hippocampal volume to the sum of the hippocampal and inferior lateral ventricle volumes in each hemisphere, left and right hippocampal occupancy scores were averaged, and then normalized for age and sex.

p values for PACC, Digit Symbol, FCSRT, Logical Memory Delay, MMSE, CFI-PT, Average Cortical Thickness, Hippocampal Occupancy, and PET_SUVr are adjusted for age, sex, and education.
